# Ectopic thyroid tissue implantation associated with hyperthyroidism after endoscopic thyroidectomy: a case report

**DOI:** 10.3389/fsurg.2026.1850986

**Published:** 2026-08-21

**Authors:** Jianping Huang, Zhu Yu, Lv Lv

**Affiliations:** 1 Department of Thyroid Surgery, Liuzhou People's Hospital / Liuzhou People's Hospital Affiliated to Guangxi Medical University, Liuzhou, Guangxi, China; 2 Guangxi Key Laboratory of Enhanced Recovery After Surgery for Gastrointestinal Cancer, The First Affiliated Hospital of Guangxi Medical University, Nanning, Guangxi, China

**Keywords:** ectopic thyroid tissue, endoscopic thyroidectomy, hyperthyroidism, iatrogenic implantation, thyroid tissue implantation

## Abstract

**Background:**

Needle track implantation is a rare complication of endoscopic surgery. This appears to be the first reported case of ectopic thyroid tissue implantation in the anterior chest wall following endoscopic thyroidectomy, accompanied by hyperthyroidism.

**Case description:**

The patient underwent endoscopic thyroidectomy for a 5 cm left thyroid adenoma. Within one year postoperatively, she noted a chest wall mass extending from the left subclavicular region to the line connecting the left nipple, accompanied by hyperthyroidism. Following treatment with antithyroid drugs, thyroid function was well controlled and the chest wall mass ceased to enlarge. Pathological examination confirmed the left anterior chest wall mass as ectopic thyroid tissue.

**Conclusions:**

This case highlights not only the risk of tissue fragment dissemination following endoscopic thyroidectomy but also indicates that ectopic thyroid tissue, under favorable microenvironmental conditions, can preserve follicular structure and may be involved in metabolic activity.

## Introduction

Laparoscopic thyroid surgery has been widely used for the treatment of both benign and malignant thyroid diseases for several decades. Its aesthetic appeal, small incisions, and magnification capabilities have made it highly favored by surgeons. In comparison to traditional open thyroid surgery, laparoscopic thyroidectomy requires the creation of additional space, which can lead to related complications such as subcutaneous emphysema, subcutaneous hematoma, and needle tract implantation of tumor cells. This report describes the case of a young female patient who developed a chest wall mass two years after undergoing laparoscopic thyroid surgery and was diagnosed with hyperthyroidism. Postoperative pathology confirmed that the chest wall mass consisted of thyroid tissue with nodular thyroid goiter changes. Previous reports have described cases of needle tract implantation during laparoscopic or robot-assisted thyroid surgeries. To our knowledge, this is the first reported case of ectopic thyroid tissue implantation after laparoscopic surgery associated with hyperthyroidism. We present this article in accordance with the CARE reporting checklist.

## Case presentation

**Figure 1:**
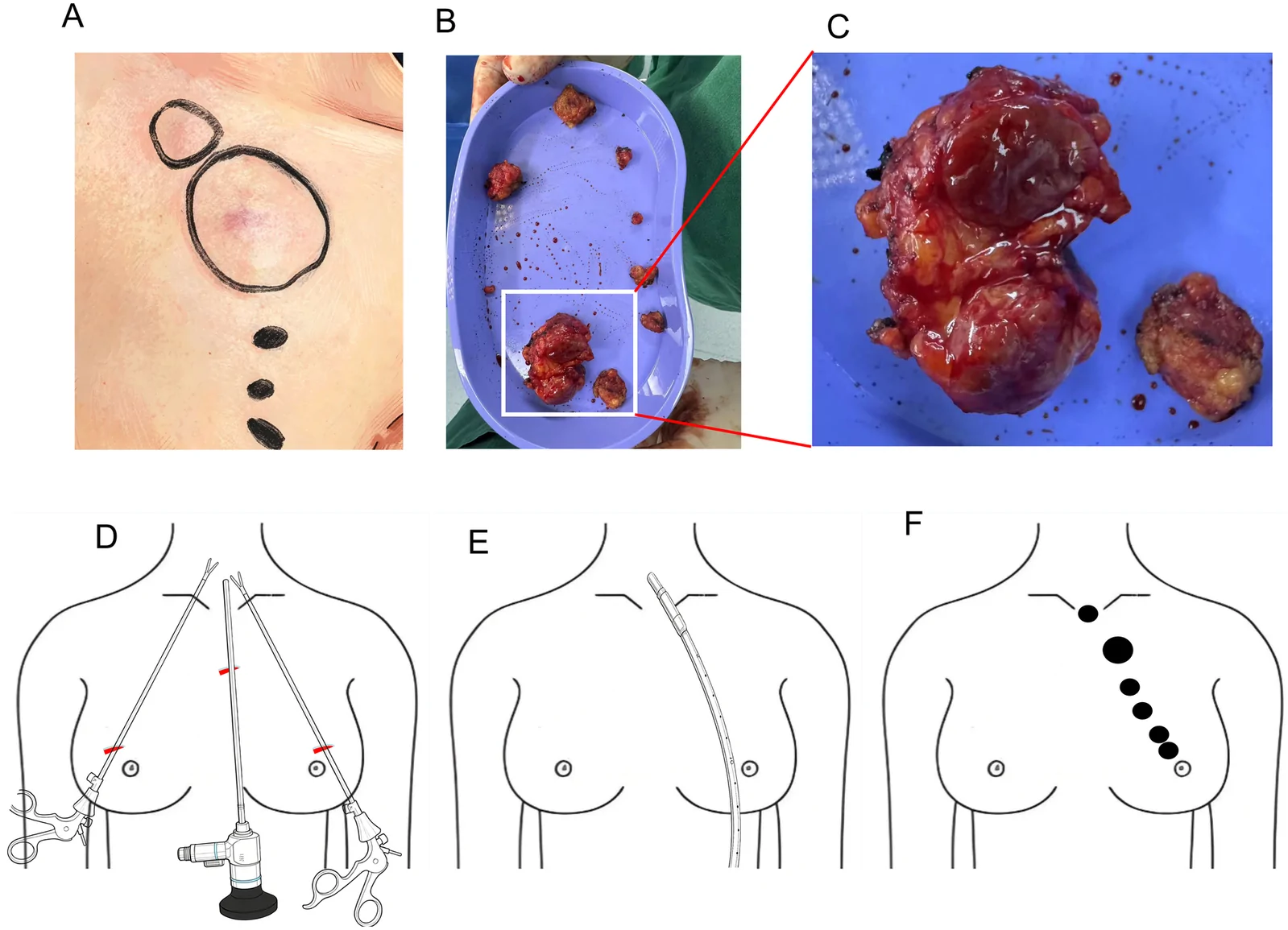
**(A)** the tumor on the left anterior chest wall of the patient is arranged in a linear pattern. **(B)** General view of the gross appearance of the left chest wall tumor. **(C)** Ultrasound image of a mass on the left chest wall. **(D)** The first surgical approach. **(E)** Placement position of the drainage tube. **(F)** The location of the growth of the chest wall tumor.

**Figure 2:**
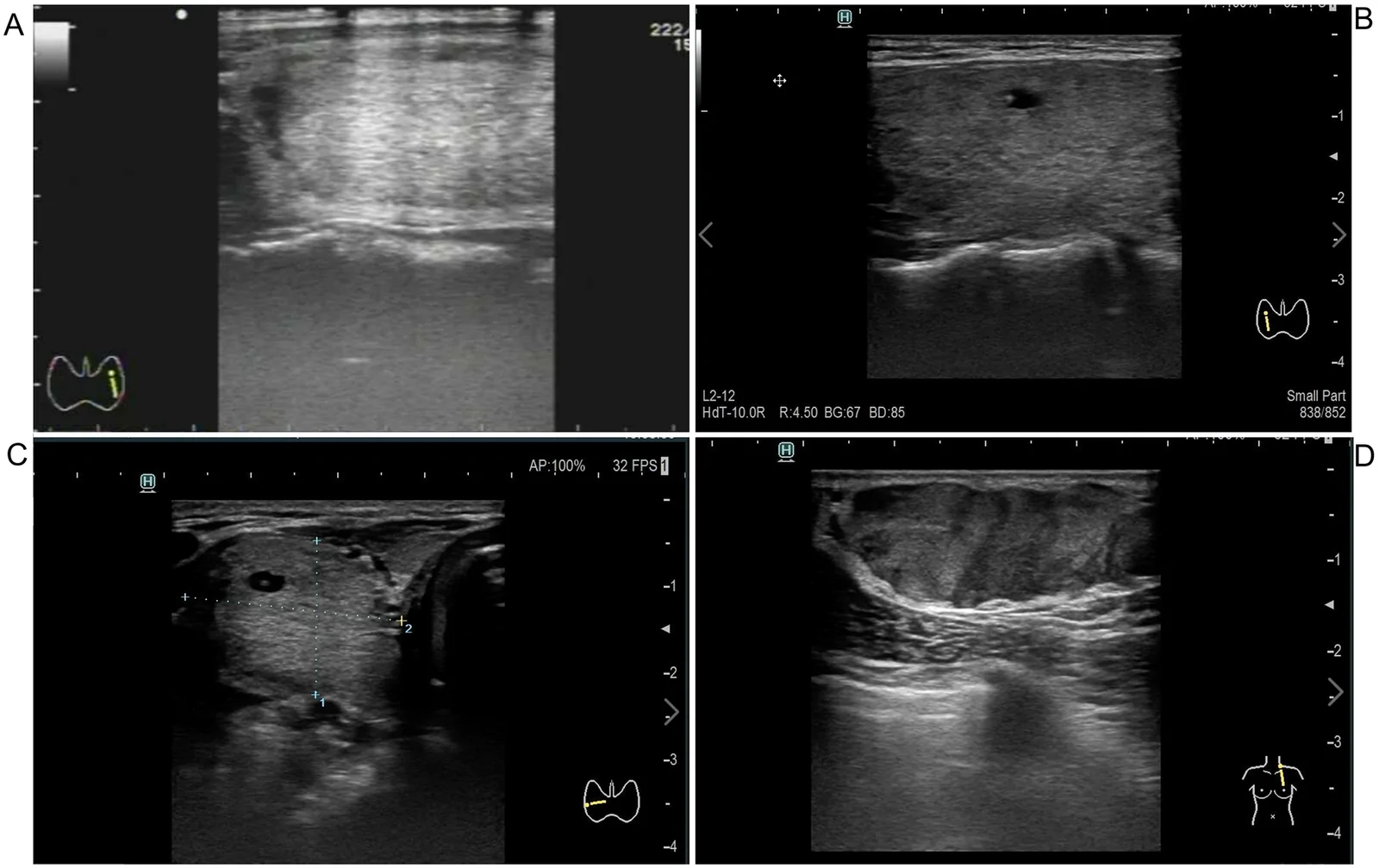
**(A)** the initial image of the left thyroid mass in the patient. **(B)** Imaging of the right thyroid gland of patient after hyperthyroidism treatment. **(C)** Imaging of the right thyroid gland of patient after hyperthyroidism treatment. **(D)** Thoracic wall mass imaging.

**Figure 3:**
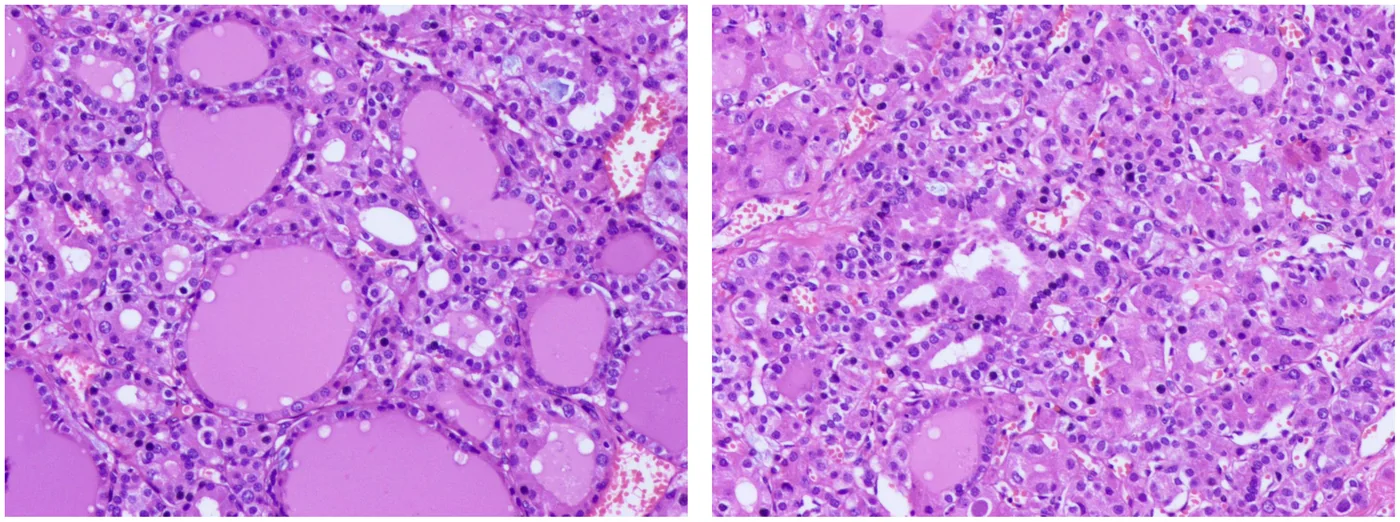
The histopathology of the chest wall mass (HE staining,   ×  100 magnification) revealed the presence of ectopic thyroid components, with active proliferation of follicular epithelium.

**Figure 4:**
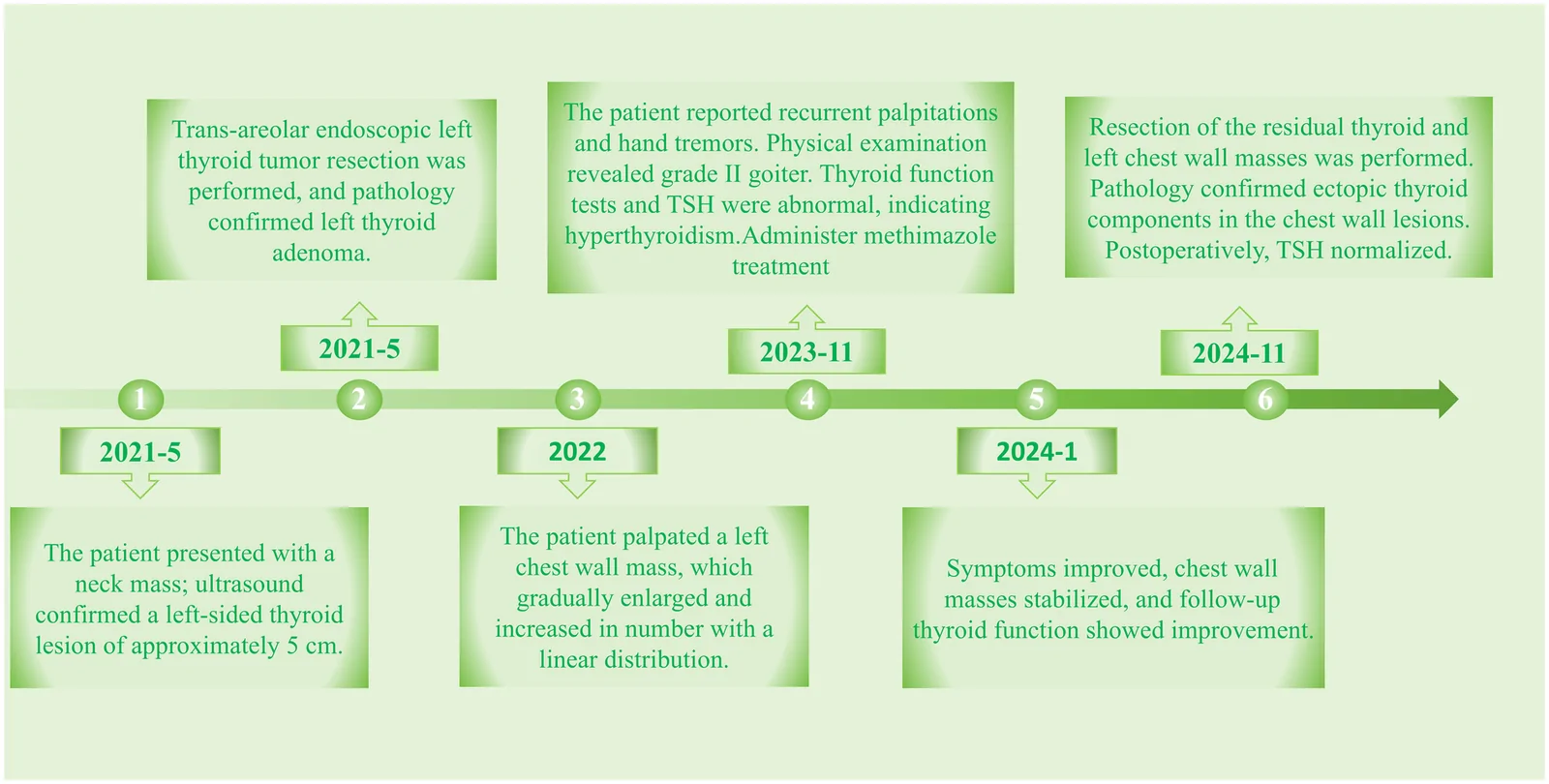
Course of the disease and timeline of diagnosis and treatment.

A 29-year-old female patient was admitted for examination due to a mass in the left neck and anterior chest wall, accompanied by hyperthyroidism. The patient had undergone laparoscopic left thyroid tumor resection via a trans-areolar approach in 2021 for a “left benign thyroid tumor,” with the tumor measuring 5 cm. Preoperative thyroid function and TRAb levels were within normal limits. At that time, TGAB and TM-AB tests were also conducted. (Table 1) Left thyroid tumor enucleation was performed with intact specimen extraction without capsular rupture. The tumor was placed in a specimen bag, fragmented, and removed through the left port. No bag rupture was observed. A spiral closed-suction drain was placed in the left port site postoperatively. (Figures 1D, E) The pathology report indicated a left thyroid adenoma with fibrosis in the surrounding thyroid areas and lymphoid tissue hyperplasia in the stroma. The patient did not follow up on time postoperatively and reported feeling irregularity in the left tunnel after the drainage tube was removed. One year after the surgery, the patient noticed a subcutaneous mass beneath the left clavicle, which gradually increased in size. Subsequently, new masses appeared along the line from the left clavicle to the left nipple and continued to enlarge. In November 2023, the patient was diagnosed with hyperthyroidism due to recurrent palpitations and hand tremors. On examination, the thyroid was enlarged to grade II, and multiple soft, well-defined masses were noted on the anterior chest wall. At that time, thyroid function tests showed: T3 3.75 nmol/L, T4 192.91 nmol/L, FT3 12.98 pmol/L, FT4 24.69 pmol/L, TSH <0.008 mIU/L, and TRAB 33.06 IU/L. The patient was treated with methimazole, after which hormone levels decreased, symptoms improved, and the left anterior chest wall mass did not continue to enlarge, thyroid ultrasound showed a plump residual thyroid with a smooth surface and diffuse changes, compatible with post-treatment changes in hyperthyroidism. (Figures 2C, D) Subsequent thyroid function test results are shown in Table 1. Before the surgery, our test showed that TPO-AB was positive. In November 2024, a physical examination at our hospital revealed several masses distributed along the line from the neck to the left areola on the left anterior chest wall, with clear boundaries, firm texture, and moderate mobility (Figure 1A). Ultrasound examination revealed multiple solid masses in the left breast adipose layer, the largest measuring approximately 40*29*15 mm. The masses had clear boundaries, moderate echogenicity, uneven internal echotexture, and partial fusion (Figure 2D). Surgical removal of the chest wall mass was planned. During surgery, multiple round masses were observed in the left anterior chest wall, the largest measuring approximately 4 cm in diameter. The masses appeared to be encapsulated, with a firm texture and a pale pink color (Figures 1B, C). Postoperative pathology: 1. The majority of the right thyroid was consistent with nodular thyroid goiter, with cyst formation in some areas. 2. The left chest wall mass was found to contain ectopic thyroid tissue within fibrofatty tissue, presenting as nodular thyroid goiter with follicular adenoma-like changes and active follicular epithelial hyperplasia. Immunohistochemistry results: CD56 (+), CyclinD1 (+), CK19 (focal +), M.C (focal +), P53 (scattered few +), Ki-67 (+, approximately 2%), CD34 (+, indicating vasculature); “Slide 7” CD34 (+, indicating vasculature) (Figure 3). One month post-surgery, follow-up thyroid function tests showed TSH 8.9 mIU/L, with all other levels within normal range. (Table 1). The detailed clinical timeline of the patient's disease progression, diagnostic evaluation, and therapeutic interventions is summarized in Figure 4.

**Table 1:** Test results of thyroid function and related antibodies.

Parameter	2021−5	2023−11	2024–1	2024–11	2024–12
T3 (nmol/L）	1.23	3.75	1.88	1.40	1.26
T4 (nmol/L）	91.95	192.91	92.84	69.07	70.71
FT3 (pmol/L）	3.88	12.98	5.12	4.54	3.90
FT4 (pmol/L)	11.75	24.69	10.48	10.21	9.96
TSH (mIU/L)	1.508	<0.008	<0.008	6.176	8.900
TRAb (IU/L)	<0.80	33.06	33.06	23.51	-
TGAB（%）	4.41	-	-	-	-
TM-AB（%）	3.53	-	-	-	-
TPO-AB(U/mL)	-	-	-	＞1,300	-

## Discussion

In recent years, advances in ultrasound technology and the standardization of fine-needle aspiration (FNA, Fine needle aspiration) have markedly improved the early diagnosis rate of thyroid cancer. Differentiated thyroid carcinoma (DTC, Differentiated thyroid carcinoma) accounts for more than 90% of cases, with an overall favorable prognosis (1). Surgery remains the cornerstone of thyroid cancer treatment. Since the first report of alternative approaches for neck surgery, endoscopic thyroidectomy has developed rapidly over the past decades. By employing concealed approaches such as trans-sternal-breast, axillary, or transoral access, this technique achieves tumor resection while offering the dual advantages of minimal invasiveness and cosmetic benefit (2). Although endoscopic thyroidectomy has been widely accepted for its outstanding cosmetic outcomes, specific complications such as carbon dioxide embolism, skin burns, and needle track implantation warrant vigilance (3). Needle track implantation, though rare, can have severe consequences. It typically occurs at puncture sites, within subcutaneous tissues, or even at distant locations, and is most often observed in cases of malignant thyroid tumors (4–7). The uniqueness of this case lies in what appears to be the first detailed report of benign thyroid tissue implantation in the anterior chest wall following a trans-sternal-breast endoscopic approach, where the implanted tissue may retain functional activity and potentially influence thyroid function.

The underlying mechanisms of needle track implantation remain incompletely understood. This case suggests that the drainage tube may serve as a vector for implantation, which differs from traditionally recognized causes such as direct seeding at the tunnel entry site, contamination of trocars, or the “chimney effect.” It highlights an additional factor that warrants attention during surgery (8). After the first surgery, the patient developed linearly arranged masses in the left chest wall, confined to one side. Their distribution partially corresponded to the trocar pathway. Despite strict adherence to tumor-free surgical principles—such as avoiding direct tumor clamping, retrieving specimens with specimen bag, and irrigating the operative field with sterile water—cellular shedding and implantation could not be entirely prevented， as we could not guarantee that no cell detachment occurred during fragmentation of the tumor within the specimen bag or during its retrieval through the port site. The turning trajectory of the linear masses at the suprasternal notch also matched the course of the indwelling drainage tube. The patient reported a foreign body sensation after drainage tube removal. Given that a spiral-pattern negative-pressure drainage tube was used, we speculate that small fragments of thyroid tissue may have been trapped within the surrounding tissue during exudate outflow, allowing implantation and subsequent growth. However, this mechanism remains speculative rather than established.

A particularly noteworthy aspect of this case is that the implanted tissue was confirmed pathologically as thyroid tissue, which survived, proliferated, and maintained follicular architecture and function within the ectopic adipose environment. Previous reports have described nodules resembling thyroid follicular structures arising in the cervical periphery after thyroidectomy, some of which led to thyrotoxicosis. In one such case, no recurrence was observed after radioactive iodine therapyRémy Louvel et al. reported a case of needle track implantation following endoscopic thyroidectomy via an axillary approach, in which benign thyroid nodules were found along the implantation tract; however, thyroid functional status was not specified (9). Similarly, Moon Young Oh et al. reported that even after surgical excision of implanted lesions, whole-body SPECT/CT iodine imaging continued to demonstrate iodine uptake at the original implantation site (4). In this case, the implanted tissue likely originated as fragmented thyroid tissue, which subsequently developed into nodular goiter and adenoma within the chest wall adipose tissue. Alternatively, the fragments may have contained both thyroid and adenomatous tissue, which co-proliferated in the adipose layer. Regardless of the mechanism, this case underscores the potential for ectopic regeneration and proliferation of thyroid tissue.

The postoperative hyperthyroidism in this case requires differentiation among multiple etiologies, including Graves' disease, toxic multinodular goiter, autonomously functioning thyroid adenoma, Hashitoxicosis, hyperfunctioning residual thyroid tissue, and ectopic thyroid tissue implantation. Graves' disease is the most important diagnosis to exclude in this case. The patient's TRAb level was markedly elevated at 33.06 IU/L, and TPOAb was positive prior to the second surgery, both significantly exceeding the normal range. In addition, the residual right thyroid lobe was found to be enlarged to grade II during the clinical course. These findings are all highly suggestive of Graves' disease. However, *de novo* Graves' disease after thyroid surgery is relatively rare, and the onset of hyperthyroidism in this patient coincided closely with the appearance of the chest wall masses. The patient underwent left thyroid tumor enucleation with preservation of the right lobe and isthmus. We cannot completely exclude the possibility that the residual thyroid tissue developed autonomously hyperfunctioning nodules or diffuse functional enhancement. Similarly, the chest wall masses were pathologically confirmed as thyroid tissue, strongly suggesting implantation from intraoperative tissue shedding. Thyroid scintigraphy, if performed, would help further clarify the functional status and relative contribution of the residual thyroid and ectopic tissues to the hyperthyroid state. Autonomously functioning thyroid adenoma and Hashitoxicosis are considered less likely, as their clinical and pathological features are not consistent with those observed in this case. While the precise source of hyperthyroidism in this patient remains uncertain, we cannot exclude the possibility that the ectopically implanted chest wall thyroid tissue is functional. Of note, the histopathological finding of proliferative thyroid follicular epithelial cells parallels recent advances in thyroid organoid research. Vivian M.L. et al. proposed that thyroid organoid cultures derived from both mice and humans contain proliferative cells with self-renewal and differentiation capabilities. When transplanted into hypothyroid mice, they formed functional thyroid follicles. This suggests that thyroid organoid-derived cells have the potential to create new miniature organs (10). Xue-Jiao Tian et al. demonstrated that transplanting thyroid tissue blocks into the spleens of mice led to effective revascularization of the transplanted tissue within 48 h. Four weeks later, thyroid follicles resembling those of the natural thyroid had formed, completely restoring thyroid function. Ultimately, the transplanted tissue grew to a size comparable to the natural thyroid (11). However, there have been no reports to date of thyroid tissue transplanted into muscles or other sites fully regenerating.

Hypothyroidism is a common disorder, and although thyroid hormone replacement therapy can achieve satisfactory physiological effects, a substantial proportion of patients remain undertreated, resulting in reduced quality of life. However, current research on thyroid regeneration remains confined to the laboratory stage, and achieving full maturation and functionality of human thyroid organoids *in vitro* or *in vivo* continues to pose significant challenges (12). In this case, the implanted tissue within the anterior chest wall adipose layer manifested as round nodules resembling thyroid glandular tissue. The ectopic thyroid tissue may have retained function and proliferated under stimulation, yet growth ceased following antithyroid therapy. Such tissue may also maintain TSH-responsive regulation akin to the native thyroid. The most significant limitation of this report is the absence of functional imaging specifically, thyroid scintigraphy or radioiodine uptake studies which would have been essential to confirm whether the chest wall lesions possess iodine-concentrating capacity. Without such data, we cannot definitively determine the relative contribution of the ectopic thyroid tissue versus the native residual gland or a concurrent Graves' disease to the patient's hyperthyroid state. Consequently, the causal relationship between the ectopic implantation and hyperthyroidism, while strongly suggested by the anatomical distribution and pathological findings, remains unproven. Despite this limitation, we believe the case provides a valuable clinical alert regarding the possibility of functional ectopic tissue after endoscopic surgery. For future similar cases, we strongly recommend early functional imaging to accurately quantify the contribution of ectopic lesions and guide appropriate management. Based on prior experience, if the growth of the implant proves to be TSH-dependent, suppressive hormone therapy may be employed. In addition, surgical excision or radioiodine ablation may also be considered as therapeutic options (13, 14).

## Conclusions

In summary, this rare case of anterior chest wall implantation with postoperative hyperthyroidism following endoscopic thyroidectomy underscores the critical importance of surgical approach selection and meticulous tumor-free techniques. Intraoperatively, rigorous adherence to tumor-free principles is essential; postoperatively, drainage devices should be chosen with caution, and follow-up should encompass puncture sites and subcutaneous tissues as potential sites of implantation. In cases of unexplained postoperative hyperthyroidism, thyroid scintigraphy and radioiodine uptake testing are warranted to exclude ectopic thyroid tissue. This case highlights the risk of tissue fragment dissemination and suggests that ectopic thyroid tissue can maintain follicular structure and potential thyroid functional capacity in a suitable microenvironment, potentially contributing to metabolic abnormalities. Nevertheless, this phenomenon offers a novel perspective for thyroid regeneration research: elucidating the survival mechanisms of functional ectopic thyroid tissue may advance strategies for stem cell-derived thyroid cell differentiation and organoid transplantation to treat hypothyroidism, ultimately providing new avenues for achieving functional thyroid tissue regeneration.

## Data Availability

The original contributions presented in the study are included in the article/Supplementary Material, further inquiries can be directed to the corresponding author.
